# The Impact of Ultrasound-Guided Rectus Sheath Blocks on Intraoperative Anesthetic Requirements and Early Recovery in Laparoscopic Surgery: A Retrospective Cohort Study

**DOI:** 10.7759/cureus.116947

**Published:** 2026-09-25

**Authors:** Sherin Abdelhamid, Khaled Abuamra, Ahmed A Khalaf, Hatem Ibrahim, Ahmad Nabil

**Affiliations:** 1 Department of Anesthesia, Dubai Hospital, Dubai Health, Dubai, ARE

**Keywords:** analgesics, enhanced recovery after surgery, laparoscopy, nerve block, opioid, opioid-sparing anesthesia, rectus sheath block, retrospective studies

## Abstract

Background and objective

The rectus sheath block (RSB) is increasingly utilized for multimodal analgesia in abdominal surgeries. A properly administered RSB may reduce nociceptive input, thereby lowering the requirement for systemic opioids and intravenous anesthetic agents such as propofol, as well as sedative/analgesic adjuncts such as dexmedetomidine. Moreover, effective regional blockade may attenuate the surgical stress response, contributing to more hemodynamic stability and reducing the use of vasopressors like phenylephrine. This retrospective study aims to evaluate the intraoperative impact of ultrasound-guided RSB in adult patients undergoing laparoscopic abdominal surgery.

Methods

This retrospective, comparative observational study included 331 adult patients with American Society of Anesthesiologists (ASA) physical status I-III, who underwent elective laparoscopic appendectomy, cholecystectomy, or midline ventral hernia repair (including umbilical and midline incisional hernias). Patients were stratified into an intervention cohort receiving ultrasound-guided RSB (block group, n = 126) and a standard care cohort (no-block group, n = 205). As a non-randomized retrospective study, block allocation was determined by the attending anesthesiologist's discretion, largely influenced by provider preference and operative time constraints. Primary outcomes included the total intraoperative opioid dose (calculated as morphine equivalents), total intravenous anesthetic dose (propofol), and sedative/analgesic adjunct (dexmedetomidine). Secondary outcomes included vasopressor requirement (phenylephrine), time spent in the post-anesthesia care unit (PACU), sedation level at PACU admission, and duration of oxygen supplementation, which may serve as surrogate markers for oversedation related to intraoperative drug administration.

Results

Baseline demographic and surgical characteristics were well matched. The block group demonstrated a significant reduction in intraoperative opioid consumption compared to the no-block group (median: 5.0 vs. 9.0 morphine milligram equivalents, p < 0.001), with multivariable adjustment confirming a 62.4% reduction in modelled exposure (p < 0.001). However, total intraoperative doses and infusion rates of propofol, dexmedetomidine, and phenylephrine demonstrated no statistically significant differences between the cohorts. Regarding recovery, the RSB was associated with a 72% reduction in the adjusted odds of presenting to the PACU with a modified Aldrete score < 9 (OR: 0.28, p < 0.001). Prolonged oxygen supplementation (>30 min) and overall PACU time did not differ significantly.

Conclusions

In laparoscopic abdominal surgery, ultrasound-guided RSB serves as a highly effective intraoperative opioid sparing adjunct that significantly enhances immediate functional recovery and PACU discharge readiness. However, the block does not significantly reduce the intraoperative requirements for intravenous anesthetics (propofol), sedative adjuncts (dexmedetomidine), or vasopressors (phenylephrine), indicating its primary intraoperative utility lies in opioid reduction rather than comprehensive anesthetic-sparing or hemodynamic modulation.

## Introduction

The rectus sheath block (RSB) is a regional anesthetic technique increasingly utilized as part of multimodal analgesia for abdominal surgeries. It involves the injection of local anesthetic into the rectus sheath to block the anterior cutaneous branches of the lower thoracic nerves. While its effectiveness in reducing postoperative pain is well established, data on its intraoperative benefits remain limited, particularly in adults undergoing routine laparoscopic abdominal procedures [1,2].

Most existing studies have concentrated on postoperative pain scores and opioid consumption, leaving a gap in understanding RSB’s role in reducing intraoperative analgesic and anesthetic demands, especially in the context of retrospective analyses [3,4]. A properly administered RSB is believed to reduce nociceptive input from the abdominal wall, thereby lowering the requirement for systemic opioids and intravenous anesthetic agents such as propofol and dexmedetomidine [5,6]. Moreover, effective regional blockade may blunt the somatic nociceptive input, contributing to more hemodynamic stability and reducing the use of vasopressors like phenylephrine [7].

However, standardized anesthetic protocols, which often include fixed intraoperative opioid and anesthetic doses regardless of adjunct blocks, can mask the true clinical benefit of RSB. This limitation underscores the importance of retrospective evaluation, where actual intraoperative drug consumption and physiological data can be used to infer the block’s effectiveness [4,6]. Such analysis is particularly relevant for evaluating opioid-sparing effects and sedation profiles in real-world settings. This retrospective study aims to evaluate the intraoperative and early recovery impact of ultrasound-guided RSB compared to standard care in adult patients undergoing elective laparoscopic abdominal surgeries (appendectomy, cholecystectomy, and midline ventral hernia repair). The primary outcomes are the total intraoperative opioid dose (calculated as morphine equivalents) and the total intraoperative doses of the intravenous anesthetic (propofol) and the sedative-analgesic adjunct (dexmedetomidine). Secondary outcomes include intraoperative vasopressor requirements, time to tracheal extubation, post-anesthesia care unit (PACU) duration, early functional recovery (assessed via the modified Aldrete score), oxygen supplementation, postoperative analgesic requirements, and overall hospital length of stay.

## Materials and methods

This retrospective, single-center, comparative observational study was conducted at Dubai Hospital, a tertiary referral center within Dubai Health, United Arab Emirates. Following ethical approval from the Dubai Health Authority Human Research Ethics Committee (Approval No.: DSREC-10/2020_16), data were extracted from the institutional electronic health record system (Epic®, Epic Systems Corporation, Verona, WI). The study included adult patients who underwent elective laparoscopic abdominal surgeries, specifically appendectomy, midline ventral hernia repair (including umbilical and midline incisional hernias), and cholecystectomy between January 2018 and October 2021. Because of the retrospective observational design of this study, a formal a priori sample size calculation was not performed. Instead, the sample size was determined by the temporal availability of data, encompassing a consecutive sample of all eligible patients treated during the defined study period. For comparative analysis, patients were stratified into two cohorts: those who received an ultrasound-guided RSB (block group) and those who did not (no-block group).

Study population, inclusion, and exclusion criteria

The study population comprised adult patients (aged 18 years and older) with an American Society of Anesthesiologists (ASA) [8] physical status of I to III who underwent laparoscopic abdominal surgeries, specifically appendectomy, cholecystectomy, and midline ventral hernia repair (including umbilical and midline incisional hernias), and were assessed for eligibility. All procedures were performed by the general surgery team under general anesthesia. Final inclusion necessitated the availability of complete intraoperative anesthesia and electronic medical records for comprehensive analysis.

Patients were excluded from the study if they possessed an ASA physical status of IV or higher, a pre-existing opioid use disorder, or a documented history of chronic opioid therapy. Patients undergoing inguinal hernia or other non-midline abdominal wall repairs were excluded. Surgical and anesthetic exclusions encompassed intraoperative conversion to an open surgical approach, the performance of concurrent surgical procedures, or the administration of the RSB in conjunction with other regional anesthesia techniques (e.g., transversus abdominis plane (TAP) block, epidural, and neuraxial spinal anesthesia). Finally, cases with incomplete documentation regarding intraoperative anesthetic or analgesic administration were excluded from the final analysis. A total population sampling approach was applied, including all eligible cases during the study period. Because this was a retrospective, non-randomized observational study, the allocation of patients to the block or no-block cohort was not dictated by a standardized clinical protocol. The decision to perform an ultrasound-guided RSB was made on a case-by-case basis at the discretion of the primary anesthesia provider. Typical factors influencing this decision in our real-world clinical setting included the attending anesthesiologist’s preference and expertise with regional fascial plane blocks, as well as time constraints and logistical pressures in the operating room. No formal a priori sample size calculation was performed because of the retrospective design; all eligible cases were included to maximize precision. Effect sizes with 95% confidence intervals (CIs) are reported.

Standardized perioperative anesthetic management

To ensure consistency in perioperative care, a single anesthesia team and surgical consultant managed all cases. Standard ASA monitoring was applied continuously throughout the perioperative period [8]. Following fixed institutional protocols, we employed a multimodal general anesthesia approach to maximize therapeutic benefits while minimizing dose-dependent side effects. General anesthesia was induced with intravenous (IV) fentanyl (1-2 µg/kg), propofol (2-4 mg/kg), and rocuronium (0.6-1.0 mg/kg) to facilitate endotracheal intubation. Anesthesia was maintained using a volatile agent (sevoflurane or desflurane) in an oxygen-air mixture, supplemented by a continuous propofol infusion (50-150 µg/kg/min). Depth of anesthesia was objectively monitored in all cases using the Bispectral Index (BIS), with agents titrated to consistently maintain a BIS value within the surgical anesthesia range of 40 to 60.

Intraoperative analgesia was guided by hemodynamic responses utilizing incremental opioid doses. Upon induction, all patients received multimodal adjunctive analgesia consisting of IV paracetamol (15 mg/kg), parecoxib (0.1 mg/kg), and dexamethasone (0.1 mg/kg). Dexmedetomidine was administered as an initial bolus of 0.5 µg/kg, followed by a continuous maintenance infusion (0.2-0.5 µg/kg/h) adjusted according to hemodynamic tolerance and clinical requirements.

Neuromuscular blockade was continuously monitored using quantitative train-of-four (TOF) monitoring. Prior to extubation, residual neuromuscular blockade was specifically excluded and routinely reversed in all patients using a standardized combination of neostigmine (0.04-0.05 mg/kg) and atropine (0.015-0.02 mg/kg). Tracheal extubation was performed only upon meeting strict objective and clinical criteria, defined as a quantitative TOF ratio > 97%, accompanied by the return of adequate spontaneous ventilation, intact airway reflexes, and clinical signs of complete neuromuscular recovery.

Ultrasound-guided rectus sheath block technique

In the intervention cohort, the RSB was executed by the attending anesthesiologist under strict aseptic precautions following anesthetic induction and prior to surgical incision. The fascial plane block was performed under continuous ultrasound guidance using a SonoSite X-Porte ultrasound system equipped with a high-frequency (6-13 MHz) linear transducer (FUJIFILM SonoSite, Inc., Bothell, WA, USA). With the transducer oriented in a transverse plane, the rectus abdominis muscle and the posterior rectus sheath were identified. Using an in-plane, lateral-to-medial needle trajectory, a total volume of 40 mL of 0.375% ropivacaine (20 mL per side) was deposited bilaterally into the fascial plane between the posterior rectus sheath at the lateral-posterior border of the rectus muscle.

Postoperative analgesia protocol

A standardized postoperative multimodal analgesic regimen was applied uniformly across both study groups. This protocol consisted of IV paracetamol 15 mg/kg (maximum 1 g) administered every eight hours, alongside IV parecoxib 40 mg administered twice daily. Rescue analgesia, comprising IV morphine in 2-3 mg incremental doses, was strictly reserved for patients reporting severe breakthrough pain, defined as a visual analog scale (VAS) score of ≥7 out of 10.

Data collection and outcomes

Data extraction and study endpoints data were systematically extracted from intraoperative anesthesia charts and institutional electronic medical records. Baseline characteristics and procedural variables collected included patient demographics (age, sex, body mass index (BMI)), ASA physical status, pre-existing comorbidities, and the specific type and total duration of the surgical procedure.

The primary outcomes of this study were total intraoperative opioid consumption, which was standardized and converted to morphine milligram equivalents (MME), and intraoperative variables collected included continuous infusion rates and total doses of anesthetics and opioids. To ensure uniform quantification of analgesic exposure, all intravenously administered opioids were standardized to intravenous morphine equivalents (IV-MEQ) using established equianalgesic conversion factors: oxycodone 1 mg = 1 mg IV morphine; nalbuphine 1 mg = 1 mg IV morphine; tramadol 10 mg = 1 mg IV morphine; and pethidine 10 mg = 1 mg IV morphine, with IV morphine serving as the reference standard.

Secondary outcomes encompassed intraoperative vasopressor requirements (quantified by total phenylephrine dose in µg and infusion rate), the time interval from surgical completion to successful tracheal extubation, total PACU duration, and overall hospital length of stay. Additionally, the requirement for postoperative supplemental oxygen exceeding 30 minutes was recorded. Because a key secondary outcome of this evaluation was to assess overall functional recovery and systemic physiological stability rather than an isolated sedation metric, early postoperative recovery upon PACU admission was evaluated using the total aggregate score of the modified Aldrete scoring system [9]. This established tool evaluates five physiological domains (consciousness, mobility, breathing, circulation, and oxygen saturation) to generate a cumulative score ranging from 0 to 10, with an aggregate score of ≥9 serving as the standard institutional benchmark for baseline stability and PACU discharge readiness. For the purposes of this retrospective analysis, data extraction was limited solely to the total aggregate score recorded in the chart at the time of PACU admission; individual domain sub-scores were not extracted. Furthermore, because these total scores were obtained directly from routine, standard-of-care electronic medical records rather than through prospective administration of the instrument, formal copyright permission for its use was not required. Finally, postoperative analgesic outcomes evaluated included the incidence of PACU pain (defined as a pain score >0), total opioid requirements within the first 24 hours post-surgery, and the incidence of documented opioid-related complications.

Statistical analysis

Descriptive statistics were utilized to summarize patient characteristics and baseline perioperative variables. Continuous variables were tested for normality and reported as medians with interquartile ranges (IQR). Categorical variables were reported as absolute frequencies and percentages (n, %). For unadjusted bivariate comparisons between the block group and no-block group, the Mann-Whitney U test was employed for continuous variables. Categorical variables were compared using the chi-square test, or Fisher’s exact test for outcomes with small expected cell counts (e.g., opioid complications). To account for multiple testing across the primary unadjusted comparison set, p-values were adjusted using the Benjamini-Hochberg procedure, yielding false discovery rate (FDR) q-values.

To isolate the independent effect of the RSB while controlling for potential confounders, multivariable regression models were constructed. All models were a priori adjusted for age, sex, BMI, ASA status, emergency/elective status, grouped surgery type, and surgery duration. For continuous, non-negative outcomes demonstrating marked skewness or zero-inflation (e.g., intraoperative opioid exposure, extubation delay, and length of stay), robust ordinary least squares (OLS) regression was applied on the log1p scale; effect sizes for these models are presented as approximate percentage changes with 95% confidence intervals (CIs). Multivariable logistic regression was utilized for binary categorical outcomes to calculate adjusted odds ratios (OR) and 95% CIs. Statistical significance for main unadjusted tests was assessed by evaluating the FDR q-value, while a standard p-value <0.05 was considered statistically significant for multivariable models. Statistical analyses were performed using SPSS version 28.0 (IBM Corp., Armonk, NY, USA).

## Results

Baseline and perioperative characteristics

A total of 331 patients were included in the analysis, consisting of 126 in the block group and 205 in the no-block group. Although cohort allocation was inherently driven by provider discretion and logistical factors rather than a randomized clinical protocol, the resulting baseline demographic and clinical characteristics were highly comparable between the two cohorts, indicating a well-balanced distribution with minimal baseline selection bias (Table 1). There were no statistically significant differences regarding median age (34.0 vs. 31.0 years; P = 0.525), body weight (P = 0.961), BMI (P = 0.561), or proportion of female patients (43.7% vs. 45.4%; P = 0.761).

**Table 1:** Baseline demographic and clinical characteristics of the study population. Data are presented as median (interquartile range) for continuous non-parametric variables and as frequency n (%) for categorical variables. Statistical comparisons between the block and no-block cohorts were performed using the Mann-Whitney U test for continuous variables and the chi-square test for categorical variables.* P < 0.05 indicates nominal statistical significance. The FDR q-value represents the P-value adjusted for multiple comparisons using the false discovery rate method to minimize type I errors. Abbreviations: ASA, American Society of Anesthesiologists; BMI, body mass index; E/V, emergency/elective; FDR, false discovery rate.

Characteristic	Block (n = 126)	No-block (n = 205)	Test	Test statistic	P-value	FDR q-value
Age (years)	34.0 (26.0–40.8)	31.0 (25.0–42.0)	Mann–Whitney U	U = 13453.0	0.525	0.721
Body weight (kg)	71.0 (64.2–82.0)	72.0 (63.7–82.9)	Mann–Whitney U	U = 12873.0	0.961	0.991
BMI (kg/m²)	26.45 (23.88–30.23)	26.81 (24.22–30.12)	Mann–Whitney U	U = 12423.5	0.561	0.741
Female sex, n (%)	55 (43.7%)	93 (45.4%)	Chi-square	χ² = 0.093	0.761	0.845
ASA status, n (%)	-	-	Chi-square	χ² = 0.440	0.803	0.845
ASA I	47 (37.3%)	83 (40.5%)	-	-	-	-
ASA II	52 (41.3%)	83 (40.5%)	-	-	-	-
ASA III	27 (21.4%)	39 (19.0%)	-	-	-	-
E/V status, n (%)	-	-	Chi-square	χ² = 0.902	0.342	0.521
Emergency (E)	94 (74.6%)	143 (69.8%)	-	-	-	-
Elective (V)	32 (25.4%)	62 (30.2%)	-	-	-	-
Surgery type, n (%)	-	-	Chi-square	χ² = 8.881	0.031*	0.170
Appendectomy	91 (72.2%)	141 (68.8%)	-	-	-	-
Cholecystectomy	25 (19.8%)	59 (28.8%)	-	-	-	-
Incisional hernia repair	2 (1.6%)	0 (0.0%)	-	-	-	-
Umbilical hernia	8 (6.3%)	5 (2.4%)	-	-	-	-
Surgery duration (min)	78.5 (58.2–95.0)	75.0 (57.0–97.0)	Mann–Whitney U	U = 13136.0	0.794	0.845

Furthermore, the groups were well-matched in terms of preoperative ASA physical status (P = 0.803), E/V status (P = 0.342), and median surgery duration (78.5 vs. 75.0 minutes; P = 0.794). Although a nominal difference was observed in the distribution of surgery types performed (P = 0.031), which predominantly consisted of appendectomies and cholecystectomies in both cohorts, this difference did not remain statistically significant after adjusting for the FDR (FDR q = 0.170).

Intraoperative anesthetic requirements

Intraoperative pharmacologic management was largely comparable between the two cohorts (Table 2), with one notable exception. Intraoperative opioid consumption (morphine equivalents) was markedly lower in the block group compared to the no-block group (median: 5.00 vs. 9.00; P < 0.001, FDR q < 0.001). The association was also evident within the two major surgical subgroups: among appendectomy patients, opioid exposure was 3.0 (0-6.0) versus 8.0 (6.0-12.0) (P = 3.70×10⁻¹⁷), and among cholecystectomy patients, it was 6.0 (5.0-8.0) versus 10.0 (7.5-15.0) (P = 0.0021) (Figure 1). While the total administered dose of propofol was nominally higher in the block cohort (median: 481.5 mg vs. 440.0 mg; P = 0.018), this difference did not retain statistical significance after adjusting for the FDR (q = 0.117). Furthermore, there were no significant differences observed between the groups regarding the choice of volatile anesthetics, maintenance minimum alveolar concentration (MAC) percentage, or the total doses and infusion rates of rocuronium, dexmedetomidine, and phenylephrine (all P > 0.05).

**Table 2:** Intraoperative anesthetic and medication requirements according to block status. Data are presented as median (interquartile range) for continuous variables and as number n (%) for categorical variables. Statistical comparisons between the block and no-block cohorts were performed using the Mann-Whitney U test for continuous variables and the chi-square test for categorical variables. * P < 0.05 indicates nominal statistical significance. The FDR q-value represents the P-value adjusted for multiple comparisons using the false discovery rate method to minimize type I errors. Abbreviations: DES, desflurane; eq, equivalents; FDR, false discovery rate; Intra-op, intraoperative; MAC, minimum alveolar concentration; SEV, sevoflurane.

Characteristic	Block (n = 126)	No-block (n = 205)	Test	Test statistic	P-value	FDR q-value
Volatile anesthetic, n (%)	DES: 113 (89.7%); SEV: 13 (10.3%)	DES: 180 (87.8%); SEV: 25 (12.2%)	Chi-square	χ² = 0.781	0.337	0.562
Propofol total (mg)	481.5 (420.0–595.8)	440.0 (380.0–550.0)	Mann–Whitney U	U = 14919.5	0.018*	0.117
Propofol rate (mg/kg/h)	5.66 (4.13–6.79)	5.17 (4.12–6.46)	Mann–Whitney U	U = 14153.5	0.143	0.295
MAC %	4.00 (3.50–5.00)	4.00 (3.50–5.00)	Mann–Whitney U	U = 13189.0	0.744	0.845
Total rocuronium dose (mg)	50.0 (40.0–65.0)	50.0 (40.0–60.0)	Mann–Whitney U	U = 13407.5	0.394	0.565
Rocuronium infusion rate (mg/kg/h)	0.550 (0.442–0.690)	0.519 (0.413–0.679)	Mann–Whitney U	U = 13777.0	0.308	0.508
Total dexmedetomidine dose (µg)	38.52 (28.60–46.28)	34.00 (24.00–44.00)	Mann-Whitney U	U = 14548.0	0.053	0.179
Dexmedetomidine infusion rate (µg/kg/h)	0.387 (0.255–0.667)	0.367 (0.247–0.508)	Mann–Whitney U	U = 14449.0	0.069	0.191
Dexmedetomidine administration, n (%)	113 (89.7%)	174 (84.9%)	Chi-square	χ² = 1.563	0.211	0.387
Total phenylephrine dose (µg)	306.5 (0.0–906.0)	53.8 (0.0–558.0)	Mann–Whitney U	U = 14470.0	0.051	0.179
Phenylephrine infusion rate (µg/kg/h)	2.84 (0.00–10.01)	0.49 (0.00–6.27)	Mann–Whitney U	U = 14322.0	0.078	0.198
Phenylephrine administration, n (%)	70 (55.6%)	103 (50.2%)	Chi-square	χ² = 0.882	0.348	0.521
Intra-op opioid (morphine eq) mg	5.00 (0.00–7.50)	9.00 (7.50–14.00)	Mann–Whitney U	U = 5570.5	<0.001*	<0.001*

**Figure 1:**
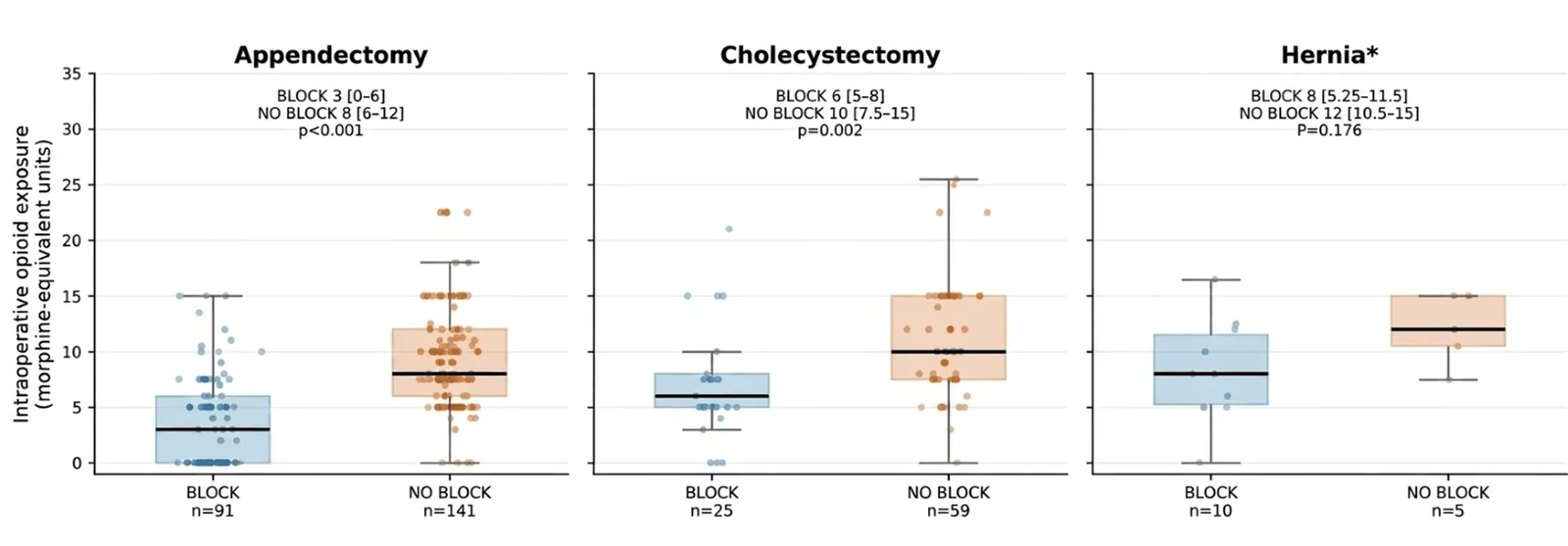
Intraoperative opioid exposure within surgical subgroups. Box-and-whisker plots illustrating intraoperative opioid consumption, expressed as intravenous morphine equivalent dose (mg), among patients undergoing appendectomy, cholecystectomy, and midline ventral hernia repair (including umbilical and midline incisional hernias). Patients receiving a rectus sheath block (block, blue) were compared with those who did not receive a block (no-block, orange). The central horizontal line represents the median value, while the box boundaries represent the interquartile range (IQR; 25th-75th percentiles). The whiskers represent the most extreme values within 1.5 times the interquartile range, and individual observations are displayed as points. Annotations above each panel report the subgroup-specific median (IQR) and corresponding Mann-Whitney U test P-value. * Incisional hernia repair cases were not displayed because of the small sample size. Abbreviations: IQR, interquartile range; n, number of patients.

Postoperative outcomes

Postoperative recovery outcomes and hospital length of stay were evaluated between the two cohorts (Table 3). Patients in the block group demonstrated a significantly higher modified Aldrete score upon PACU admission compared to the no-block group (median: 9.0 vs. 8.0; P < 0.001, FDR q < 0.001). Time to extubation was nominally longer in the block cohort by a median of 1.0 minute (P = 0.006); however, this did not retain statistical significance following FDR adjustment (q = 0.054). Similarly, a nominal difference in 24-hour postoperative opioid consumption (P = 0.049) was not significant after FDR correction (q = 0.179). There were no statistically significant differences between the groups regarding overall PACU time (P > 0.999), PACU pain scores (P = 0.060), or total length of hospital stay (P = 0.648).

**Table 3:** Postoperative recovery outcomes and hospital length of stay. Data are presented as median (interquartile range). Statistical comparisons between the block and no-block cohorts were performed using the Mann-Whitney U test for continuous non-parametric variables. * P < 0.05 indicates nominal statistical significance. The FDR q-value represents the P-value adjusted for multiple testing using the false discovery rate method to minimize type I errors. Abbreviations: FDR, false discovery rate; IQR, interquartile range; PACU, post-anesthesia care unit.

Outcome	Block (n = 126), Median (IQR)	No-block (n = 205), Median (IQR)	Median difference	Test statistic	P-value	FDR q-value
Time to extubation (min)	9.0 (7.0–14.0)	8.0 (5.0–12.0)	+1.0	U = 15209.0	0.006*	0.054
Modified Aldrete score at PACU admission	9.0 (8.0–9.0)	8.0 (8.0–9.0)	+1.0	U = 17138.5	<0.001*	<0.001*
PACU time (min) (minimum and maximum values)	38.5 (31.0–50.8)	40.0 (29.8–53.2)	−1.5	U = 12853.0	>0.999	>0.999
PACU pain score (minimum and maximum values)	0.0 (0.0–0.0) (0-7)	0.0 (0.0–0.0) (0-10)	0	U = 11914.0	0.060	0.179
Postoperative opioid within 24 h (morphine equivalent)	0.00 (0.00–0.00) (0-5)	0.00 (0.00–0.00) (0-15)	0	U = 11904.0	0.049*	0.179
Length of hospital stay (days)	2.0 (2.0–3.0)	2.0 (1.0–3.0)	0	U = 13287.5	0.648	0.822

Categorical postoperative outcomes

Analysis of categorical clinical outcomes revealed that patients in the block group had a significantly lower risk of achieving a sub-optimal modified Aldrete score (<9) at PACU admission compared to the no-block cohort (28.6% vs. 56.6%; RR = 0.50, 95% CI: 0.37-0.68; P < 0.001). While the block group demonstrated trends toward a reduced requirement for postoperative opioids within 24 hours (9.5% vs. 17.1%; P = 0.056) and a lower incidence of PACU pain (11.1% vs. 18.0%; P = 0.090), these differences did not reach formal statistical significance. Furthermore, there were no significant differences between the two groups regarding delayed extubation time >15 minutes (P = 0.183), prolonged oxygen requirement >30 minutes (P = 0.293), or the incidence of opioid-related complications (1.6% vs. 5.4%; P = 0.142) (Table 4).

**Table 4:** Categorical clinical outcomes and relative risk analysis. Data are presented as frequency n (%). Statistical comparisons between the block and no-block cohorts were performed using the Pearson Chi-square (χ2) test, except for the incidence of opioid-related complications, where Fisher’s exact test was utilized due to small expected cell counts. Risk analysis is presented as unadjusted relative risk (RR) alongside 95% confidence intervals (CIs). * P < 0.05 indicates statistical significance. Abbreviations: CI, confidence interval; PACU, post-anesthesia care unit; RR, relative risk.

Outcome	Block events, n (%)	No-block events, n (%)	Unadjusted RR (95% CI)	Test statistic	P-value
Time to extubation >15 min	22/126 (17.5%)	25/205 (12.2%)	1.43 (0.84–2.43)	χ2 = 1.776	0.183
Modified Aldrete score at PACU admission <9	36/126 (28.6%)	116/205 (56.6%)	0.50 (0.37–0.68)	χ2 = 24.661	<0.001*
Incidence of PACU pain (score >0)	14/126 (11.1%)	37/205 (18.0%)	0.62 (0.35–1.09)	χ2 = 2.882	0.090
Oxygen requirement >30 min	15/126 (11.9%)	33/205 (16.1%)	0.74 (0.42–1.31)	χ2 = 1.106	0.293
Postoperative opioid requirement within 24 h	12/126 (9.5%)	35/205 (17.1%)	0.56 (0.30–1.03)	χ2 = 3.651	0.056
Incidence of opioid-related complications	2/126 (1.6%)	11/205 (5.4%)	0.30 (0.07–1.31)	— (Fisher’s exact test)	0.142

Multivariable adjusted clinical outcomes

To account for potential confounding variables, a multivariable regression analysis was performed adjusting for age, sex, BMI, ASA physical status, emergency/elective status, grouped surgery type, and surgical duration (Table 5). Continuous outcomes with marked skewness were modeled utilizing robust OLS regression on the log1p scale, with effects reported as approximate percentage changes. Binary outcomes were evaluated using multivariable logistic regression to generate adjusted ORs.

**Table 5:** Multivariable-adjusted associations between rectus sheath block and clinical outcomes. All models were adjusted for age, sex (reference category: male), body mass index (BMI), American Society of Anesthesiologists (ASA) physical status (reference category: ASA I), emergency/elective status (reference category: elective), grouped surgery type (reference category: appendectomy), and surgery duration. The primary predictor, rectus sheath block, was coded as a binary variable (reference category: no-block). Continuous non-negative outcomes with marked skewness were modeled using robust ordinary least squares (OLS) regression on the log1p scale; effect sizes are therefore presented as approximate percentage changes with 95% confidence intervals (CI). Binary outcomes were evaluated using multivariable logistic regression, with effects presented as adjusted odds ratios (OR). * P < 0.05 indicates statistical significance. Abbreviations: CI, confidence interval; OLS, ordinary least squares; PACU, post-anesthesia care unit; OR, odds ratio; h, hours; min, minutes.

Outcome	Model	N	Adjusted block effect (95% CI)	P-value	Interpretation
Intraoperative opioid, morphine equivalent (mg)	Robust OLS, log1p	331	−62.4% (−69.0% to −54.4%)	<0.001*	Block was associated with an approximately 62% lower modeled intraoperative opioid exposure.
Time to extubation (min)	Robust OLS, log1p	331	+21.5% (+7.6% to +37.2%)	0.002*	Block was associated with a longer modeled extubation interval.
Prolonged extubation (> 15 min)	Logistic regression	331	OR 1.51 (0.81–2.82)	0.192	The odds of >15-minute delayed extubation were not significantly different after adjustment.
Modified Aldrete score at PACU admission	Robust OLS	331	+0.56 points (+0.31 to +0.81)	<0.001*	Block was associated with a higher modified Aldrete score.
Modified Aldrete score at PACU admission <9	Logistic regression	331	OR 0.28 (0.17–0.47)	<0.001*	Block was associated with substantially lower adjusted odds of an Aldrete score <9.
PACU pain >0	Logistic regression	331	OR 0.58 (0.30–1.13)	0.108	Block was associated with lower estimated odds of PACU pain, but the association was not statistically conclusive.
PACU time (min)	Robust OLS, log1p	331	−2.6% (−11.8% to +7.6%)	0.601	No clear adjusted association with PACU duration was observed.
Oxygen requirement >30 min	Logistic regression	331	OR 0.68 (0.33–1.39)	0.290	No clear adjusted association with prolonged oxygen requirement was observed.
Postoperative opioid within 24 h	Logistic regression	331	OR 0.54 (0.27–1.10)	0.090	Block was associated with lower estimated odds of postoperative opioid use, but the CI included the null.
Length of hospital stay (days)	Robust OLS, log1p	331	−0.8% (−10.1% to +9.5%)	0.867	No clear adjusted association with length of stay was observed.

After adjustment, the RSB remained independently associated with a significant intraoperative opioid sparing effect. Patients receiving the block experienced an estimated 62.4% reduction in modeled intraoperative opioid exposure (95% CI: −69.0% to −54.4%; p = <0.001). Regarding immediate recovery metrics, the multivariable model revealed a statistically significant, albeit clinically minor, increase in the extubation interval. The block cohort was associated with a 21.5% longer modeled extubation time (95% CI: +7.6% to +37.2%; p = 0.002). However, when evaluated categorically, the odds of a clinically prolonged extubation exceeding 15 minutes were not significantly increased (OR: 1.51, 95% CI: 0.81-2.82; p = 0.192).

Despite the slight delay in extubation time, the quality of immediate postoperative recovery was significantly superior in the intervention group. The block was associated with a +0.56-point increase in the modified Aldrete score at PACU admission (95% CI: +0.31 to +0.81; p = <0.001). Consequently, the block conferred a substantial protective effect against poor early functional recovery, yielding a 72% reduction in the adjusted odds of presenting to the PACU with an Aldrete score less than 9 (OR: 0.28, 95% CI: 0.17-0.47; p = <0.001).

The block demonstrated trends toward reduced odds of PACU pain (OR = 0.58; P = 0.108) and postoperative opioid use within 24 hours (OR = 0.54; P = 0.090), though these did not reach statistical significance. There were no clear adjusted associations regarding overall PACU time, prolonged oxygen requirement, or hospital length of stay (all P > 0.05).

## Discussion

Comparability of cohorts and intraoperative dynamics

Analysis of baseline demographic and clinical characteristics revealed a well-balanced distribution between the RSB and standard care cohorts, providing a robust framework for evaluating the primary endpoints. The comparability of critical covariates such as BMI, ASA physical status, and operative duration significantly minimizes the confounding bias inherent in observational research. Although a nominal baseline imbalance was noted in the distribution of specific surgery types, this potential confounder was methodologically addressed by adjusting for grouped surgical procedures across all multivariable analyses. Consequently, the observed improvements in clinical endpoints, particularly regarding reduced intraoperative opioid consumption and enhanced early recovery metrics, can be more reliably attributed to the analgesic efficacy of the RSB.

Notably, the implementation of the RSB did not result in a statistically significant reduction in intraoperative anesthetic requirements. MAC parameters and the rates of propofol and dexmedetomidine infusion remained consistent regardless of block status. This observation aligns with established regional anesthesia literature regarding fascial plane blocks. While RSBs are highly efficacious for interrupting somatic afferent nerve transmission from the anterior abdominal wall (via the ventral rami of the 7th to 12th thoracolumbar nerves), they do not block visceral pain pathways. Consequently, surgical manipulation of intra-abdominal organs during appendectomies and cholecystectomies still necessitates an adequate depth of general anesthesia. The lack of an intraoperative "anesthetic-sparing" effect highlights that the primary clinical utility of the RSB lies in the postoperative phase of recovery, targeting somatic incisional pain [10].

Furthermore, the perioperative hemodynamic profiles of both groups were comparable, as evidenced by the similar requirements for phenylephrine. A well-documented limitation of central neuraxial techniques (e.g., thoracic epidurals) is the induction of a sympathetic blockade, frequently resulting in hypotension that requires vasopressor support [11]. In contrast, fascial plane blocks like the RSB offer targeted somatic analgesia without precipitating central sympathectomy [12]. Our findings corroborate the strong safety and stability profile of the RSB, demonstrating that its application does not impose additional hemodynamic burdens on the patient or require increased vasopressor utilization by the anesthesia provider.

Opioid sparing efficacy and early recovery

Our results highlight the substantial intraoperative opioid sparing effect of the RSB. Patients in the block cohort required significantly fewer intraoperative morphine equivalents compared to standard care. This aligns closely with the core principles of Enhanced Recovery After Surgery (ERAS) protocols, which advocate for regional anesthetic techniques to minimize systemic opioid administration. Our findings are supported by multiple clinical trials demonstrating that fascial plane blocks, including the RSB and TAP blocks, effectively obturate somatic afferent signals from the anterior abdominal wall, thereby reducing the intraoperative nociceptive burden [6,13,14].

The clinical benefit of this intraoperative opioid reduction is reflected in the significantly improved modified Aldrete scores observed in the block group. Systemic opioids are universally associated with dose-dependent central nervous system depression, respiratory depression, and delayed awakening. By relying on regional techniques to provide somatic analgesia, the block cohort likely experienced less residual opioid-induced sedation, allowing for brisker emergence and better immediate physiological scores in the PACU, a finding corroborated by literature advocating for regional techniques in ambulatory abdominal surgeries [15].

Postoperative pain profiles

Interestingly, this analysis found no statistically significant difference in postoperative PACU pain scores or 24-hour postoperative opioid consumption between the cohorts, with both groups reporting median values of zero. This contrasts with several previous studies that report a pronounced reduction in postoperative pain scores and 24-hour opioid requirements following RSB administration [16,17]. For instance, a randomized trial demonstrated a 40% reduction in 24-hour PCA (patient-controlled analgesia) opioid demand in RSB cohorts following laparoscopic surgeries [16].

The lack of difference in the postoperative variables can likely be attributed to specific factors. First, the zero-inflated nature of the postoperative opioid and pain data suggests that patients in both cohorts were overwhelmingly comfortable. This may be due to the aggressive utilization of a standardized multimodal, non-opioid analgesic regimen (e.g., scheduled acetaminophen, nonsteroidal anti-inflammatory drugs (NSAIDs), dexmedetomidine, and dexamethasone) in both groups, which successfully mitigated postoperative pain even without the block. Second, the higher doses of intraoperative opioids administered to the no-block group (median: 9.0 MME) likely provided residual systemic analgesia that carried over into the PACU phase, effectively masking the immediate postoperative analgesic superiority of the RSB. Finally, some studies argue against the routine use of RSB for certain minimally invasive abdominal procedures, suggesting that surgical site local anesthetic infiltration by the surgeon provides non-inferior postoperative analgesia to ultrasound-guided blocks, which could explain the parity in our 24-hour outcomes [18,19].

In conclusion, while the RSB did not statistically alter 24-hour postoperative opioid demand in this specific clinical pathway, likely due to robust multimodal analgesia, it successfully served as an intraoperative opioid sparing modality that facilitated superior early PACU recovery scores.

Postoperative recovery

The categorical data strongly reinforce the continuous outcomes previously reported, further establishing the role of the RSB in accelerating immediate postoperative recovery. The highly significant 50% relative risk reduction (RR = 0.50) in patients presenting with an Aldrete score at PACU admission of less than 9 highlights the physiological advantages of an opioid sparing intraoperative technique. Patients requiring less systemic analgesia intraoperatively are less prone to residual sedation and respiratory depression. This aligns with the broader consensus in the regional anesthesia literature, which advocates for fascial plane blocks to expedite PACU discharge criteria and streamline ambulatory surgical pathways [20,21].

A critical focal point of our analysis is the borderline statistical significance observed in the reduction of both early PACU pain (p = 0.090) and the need for any postoperative opioids within 24 hours (p = 0.056). While the p-values narrowly missed the conventional 0.05 threshold, the point estimates suggest a substantial clinical benefit, specifically, a 44% relative risk reduction (RR = 0.56) in the likelihood of requiring 24-hour postoperative opioids. This finding is consistent with several prospective studies that have established the analgesic efficacy of RSB for midline and paramedian incisions [1,22]. It is highly probable that our strict adherence to a baseline multimodal analgesic protocol (including NSAIDs and acetaminophen) in both cohorts created a "floor effect," thereby reducing the overall incidence of postoperative opioid use and rendering our study relatively underpowered to detect a statistically definitive difference in these specific categorical endpoints [1].

Conversely, some literature cautions that the clinical duration of a single shot fascial plane block is inherently limited, often regressing before the 24-hour mark, which could also explain the attenuation of significance at later postoperative intervals [23]. Furthermore, while there was a directional trend toward fewer opioid related complications in the block group (1.6% vs. 5.4%), the low overall event rate precluded statistical significance.

In summary, these categorical outcomes validate the RSB as a highly effective adjunct for optimizing immediate functional recovery (modified Aldrete score at PACU admission). While robust multimodal analgesia may mask the isolated long-term statistical impact of the block on 24-hour opioid consumption, the favorable clinical trends support its continued inclusion in enhanced recovery protocols.

Confirmation of opioid sparing efficacy and the extubation paradox

The multivariable analysis definitively isolates the clinical impact of the RSB, controlling for critical covariates such as surgical duration and patient comorbidities. The adjusted data confirm that the RSB is an independent predictor of drastically reduced intraoperative opioid requirements (a 62% reduction). This finding solidifies the RSB as a highly effective core component of an opioid sparing intraoperative anesthetic pathway, strongly aligning with modern ERAS principles [24,25].

An intriguing finding in our adjusted model is the "extubation paradox." The block group demonstrated a statistically significant 21.5% increase in extubation time yet exhibited significantly superior functional recovery upon PACU arrival (OR of 0.28 for modified Aldrete score at PACU admission < 9). It is crucial to contextualize the clinical relevance of this extubation delay. Given the median baseline extubation time of eight minutes in our cohort, a 21.5% increase translates to less than two additional minutes. This fractional delay, while statistically captured by the robust log1p OLS model, is clinically negligible, corroborated by the lack of significant difference in the binary outcome of delayed extubation > 15 minutes.

Interestingly, the RSB group experienced a slight delay in extubation time despite a significant reduction in intraoperative opioid consumption. This finding may be explained by the systemic absorption of the large volumes of local anesthetic required for fascial plane blocks, which can exert mild central nervous system depressant effects; this systemic "lidocaine-like" effect can subtly prolong emergence [26]. This delayed extubation can be attributed to both procedural and pharmacological factors. Performing the ultrasound-guided block after induction increases the total duration of anesthetic exposure. Furthermore, our data demonstrate higher intraoperative propofol consumption in the block group. To compensate for the lack of intraoperative opioids, anesthesiologists likely maintained a deeper hypnotic plane. The combination of prolonged anesthetic time and a higher cumulative dose of propofol requires a longer context-sensitive half-time for clearance, explaining the delayed extubation [27,28]. Crucially, however, the superior Aldrete scores indicate that once these patients are extubated, their quality of emergence is exceptionally high. By circumventing heavy intraoperative opioid loading, patients bypass the severe respiratory depression and prolonged somnolence commonly seen with traditional general anesthesia, arriving in the PACU alert and physiologically stable [29,30].

Sustained analgesia in the context of multimodal floor effects

Consistent with our unadjusted findings, the multivariable model showed that the RSB's association with 24-hour postoperative opioid consumption and PACU pain trended toward benefit (ORs of ~0.55) but did not achieve statistical significance (p > 0.05). Some contemporary literature supports this attenuation of benefit, noting that single-shot fascial plane blocks primarily cover the most acute phase of incisional nociception and frequently regress within 12 to 18 hours, diminishing their comparative impact on cumulative 24-hour metrics [24].

Conversely, other trials report sustained 24-hour opioid reductions following RSBs [16]. We hypothesize that the lack of statistical significance in our cohort is driven by an institutional "floor effect." Aggressive adherence to a non-opioid, multimodal analgesic backbone comprising scheduled acetaminophen and NSAIDs likely managed somatic pain so effectively in both groups that the window for detecting an independent RSB effect was narrowed. Consequently, while the RSB may not revolutionize 24-hour outcomes in the presence of an already optimized multimodal regimen, it undeniably optimizes the immediate transitional phase from the operating room to the PACU.

A notable limitation of this study is its retrospective, real-world design, which precludes the evaluation of the RSB in isolation. Because our institutional protocol mandates scheduled multimodal analgesia (including NSAIDs and paracetamol) for all eligible patients, the independent analgesic duration of the RSB is inevitably masked by these systemic agents. Future prospective, randomized trials with placebo controls would be required to evaluate the isolated physiological duration of the RSB, though such designs face ethical challenges in the era of standard multimodal pain management.

## Conclusions

This retrospective analysis demonstrates that ultrasound-guided RSB during laparoscopic abdominal surgeries serves as a highly effective intraoperative opioid sparing modality. While RSB did not decrease the requirement for intravenous hypnotics, inhalational agents, or intraoperative vasopressors, it maintained a robust hemodynamic safety profile without the hypotensive risks associated with neuraxial techniques. The primary clinical benefit of RSB was observed during immediate postoperative recovery; by circumventing heavy intraoperative systemic opioid loading, patients experienced significantly less residual sedation and achieved superior modified Aldrete scores upon PACU admission. Although the aggressive use of baseline multimodal analgesia likely created a floor effect that attenuated differences in 24-hour postoperative opioid consumption, this study validates RSB as a critical component of ERAS pathways. RSB successfully shifts the analgesic burden away from systemic opioids, optimizing patient safety and physiological stability during the critical transition from the operating room to the PACU.
